# Phytochemical and Nutritional Quality Changes During Irrigation and Postharvest Processing of the Underutilized Vegetable African Nightshade

**DOI:** 10.3389/fnut.2020.576532

**Published:** 2020-11-16

**Authors:** Dharini Sivakumar, Anh Dao Thi Phan, Retha M. Slabbert, Yasmina Sultanbawa, Fabienne Remize

**Affiliations:** ^1^Department of Crop Sciences, Phytochemical Food Network Research Group, Tshwane University of Technology, Pretoria, South Africa; ^2^Australian Research Council (ARC) Industrial Transformation Training Centre for Uniquely Australian Foods, Queensland Alliance for Agriculture and Food Innovation, The University of Queensland, Brisbane, QLD, Australia; ^3^Department of Horticulture, Tshwane University of Technology, Pretoria, South Africa; ^4^UMR QualiSud, Université de La Réunion, CIRAD, Université Montpellier, Montpellier SupAgro, Université d'Avignon, Sainte Clotilde, France

**Keywords:** traditional leafy vegetables, polyphenols, antioxidants, minerals, postharvest processing

## Abstract

Underutilized or traditional leafy vegetables are grown in the wild and cultivated. They are consumed as nutritional accompaniments to staples, either raw (fresh), cooked, or in a dried form, through custom, habit, and tradition. These traditional leafy vegetables are natural rich sources of phytochemicals and nutritional compounds. Over time, the keenness for consumption of traditional vegetables has become less popular. Poor nutrient diets are the main cause of mortality and morbidity, especially in developing countries, where the problem is predominant due to poverty. Consumption of traditional vegetables can assist in the prevention of chronic disease development, as they contain various bioactive compounds that exhibit multiple health benefits. Traditional leafy vegetables play a vital role in combatting hunger, food insecurity, and malnutrition, and most are suitable for food intervention programs. African nightshade (*Solanum* family) is one such commonly consumed traditional leafy vegetable. During dry seasons, communities often face shortages of vegetables; thus, the preservation of edible leaves is one strategy to help overcome this problem. The adoption of solar drying and fermentation are traditional methods to extend the availability of African nightshade vegetables. Additionally, the agronomy practices and postharvest processing methods affect the phytochemicals and nutritional compounds of African nightshade accessions. This mini-review provides information on changes in phytochemicals, nutrition, and antinutritive compounds with different postharvest processing methods and irrigation. The review provides the justification to promote the cultivation for consumption, by identifying the potential African nightshade accessions that are rich in phytonutritional compounds. This mini-review summarizes and discusses the major information on (i) the micro- and macronutrients present in *Solanum retroflexum*, the most commonly consumed nightshade species compared with other traditional vegetables in Southern Africa, (ii) the composition of phytochemical compounds present in different nightshade accessions, (iii) the impact of irrigation on phytochemical composition in different nightshade species, and (iv) the impact of postharvest processing on phytochemicals and antinutritive compounds in *S. retroflexum*. Inclusion of African nightshade, especially *S. retroflexum*, with the main staple foods can improve protein, iron, and calcium levels in daily diets, which will help to improve people's health and well-being.

## Introduction

Consumer preference for the intake of fruit and vegetables in the daily diet is increasing, and the World Health Organization (1, 2) recommends a minimum of 400 g of fruit and vegetables, or five portions, per day, excluding starchy tubers. The United States Department of Agriculture (USDA) guidelines (2011) (3) state that an individual must consume at least one cup (~237 g) of raw or cooked vegetables or two cups of raw leafy greens daily. In developing countries, particularly Africa and Asia, consumers need to meet the minimal requirement of caloric values, because micronutrient deficiency, referred to as hidden hunger, is prevalent (4). Micronutrient deficiency is due to a lack of dietary intake of calcium (Ca), iron (Fe), zinc (Zn), potassium (K), magnesium (Mg), iodine (I), copper (Cu), and selenium (Se). Additionally, vitamin A deficiency remains a common health-associated problem in South Asia and sub-Saharan Africa (5). Women of reproductive age (≥15–49 years) in Ethiopia, Kenya, Nigeria, and South Africa suffer from anemia (18–51%), iron deficiency (9–18%), and iron deficiency anemia (10%), as well as vitamin A (4–22%), iodine (22–55%), zinc (34%), and folate (46%) deficiency (6). Consequently, it is worth including underutilized African leafy vegetables in a diet diversification strategy for the sub-Saharan African population to fight against hidden hunger (7). The most commonly grown traditional African leafy vegetables in the sub-Saharan African region are of the *Amaranthus* species: wild mustard (*Brassica* spp.), African nightshade (*Solanum* spp.), sweet potatoes (*Ipomoea batatas*), spider flower (*Cleome gynandra*), Jew's mallow (*Corchorus olitorius* and *Corchorus tridens*), cowpeas (*Vigna unguiculata*), pumpkins (*Cucurbita pepo, Cucurbita maxima*, and *Cucurbita moschata*), melons (*Citrullus lanatus* and *Cucumis melo*), and balsam pear (*Momordica balsamina*). Of these, wild mustard and African nightshade are widely consumed (7). Once cooked, the leaves are an accompaniment to the staple starch-based maize meal and tomato relish; sometimes the leaves are fermented with milk. These food preparation methods help reduce the bitter-tasting compounds in the leaves derived from anti-nutrients, such as solanaceous glycoalkaloids (8).

**Figure 1:**
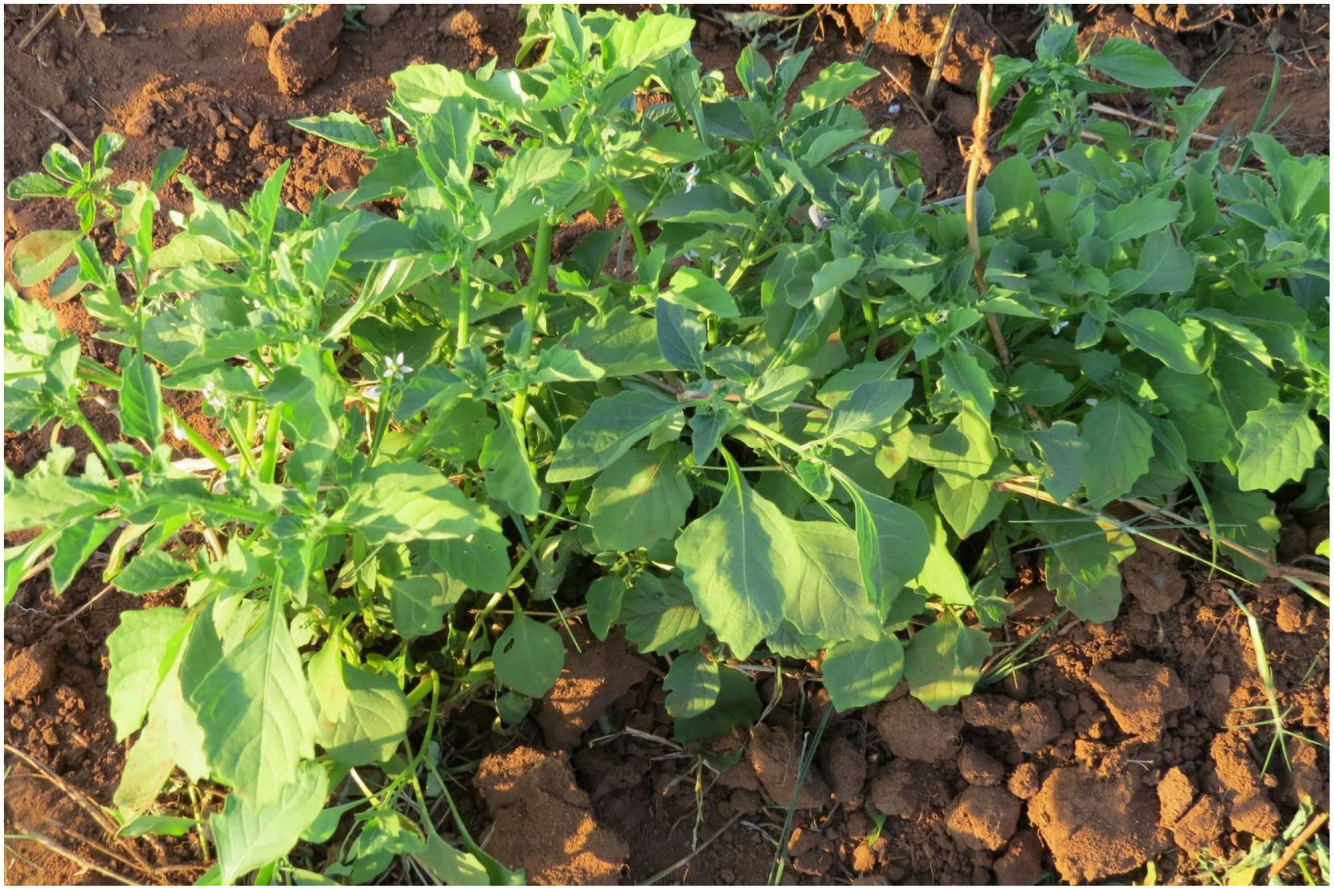
*Solanum retroflexum* (African nightshade) leafy vegetable.

Nightshade plants, propagated *via* the seeds (9), are an annual plant growing to almost 75 cm in height (9), with a simple leaf morphology—alternate margins with blunt teeth and slightly hairy. *Solanum retroflexum* (Figure 1), which is endemic to South Africa, belongs to the Solanaceae family and known as Black nightshade, or nastergal, umsobo, and muxe in the region; it is a popular leafy vegetable consumed in the Southern and Eastern parts of Africa. Other nightshade vegetables consumed in the sub-Saharan region are *S. retroflexum, Solanum americanum, Solanum nigrum, Solanum scabrum*, and *Solanum villosum* (8, 10); *S. scabrum* Mill (mnavu), a broad-leafed type of nightshade, is popular in West, Central, and East Africa (11).

In Southern Africa, subsistence farmers cultivate nightshade vegetables on a small scale and market them to generate income and improve their livelihoods. African nightshade does not need extensive fertilizer application, thrives in drought, and is less prone to pest attack; therefore, it is cost effective to produce and environmental-friendly when compared with commercial leafy vegetables (12). Currently, there are efforts to increase production, and linking these vegetables to agro-processing and the supply chain will alleviate hunger and improve nutrition and the rural economy. Unfortunately, postharvest losses of vegetables are high during marketing, primarily due to lack of cost-effective cold chain infrastructure; therefore, the traditional ways to preserve and reduce food loss are by adopting drying or fermentation technologies. Traditionally, drying was by the sun or shade drying; however, in Asian and African countries, community cooperatives have recently had cost-effective solar dryers erected. The two agro-processing technologies, solar drying, and fermentation, play a major role in facilitating the food available and sustaining food security in rural regions. These technologies are considered as recommended strategies to improve the bioavailability of micronutrients, and sometimes they can reduce the antinutritive compounds (13, 14). Considering the aforementioned, this review summarizes the research-based information on phytochemical nutritional properties of African nightshade species and the changes in phytonutritional components during irrigation or postharvest processing. In addition, this review discusses the antinutritive components in *S. retroflexum* species and changes of these compounds during postharvest processing and safety for consumption.

## Micro- and Macronutrients in Selected Nightshade Species Compared With Other Commonly Consumed Traditional Vegetables in Southern Africa and Commercial Vegetables

### Micronutrients

**Figure 2:**
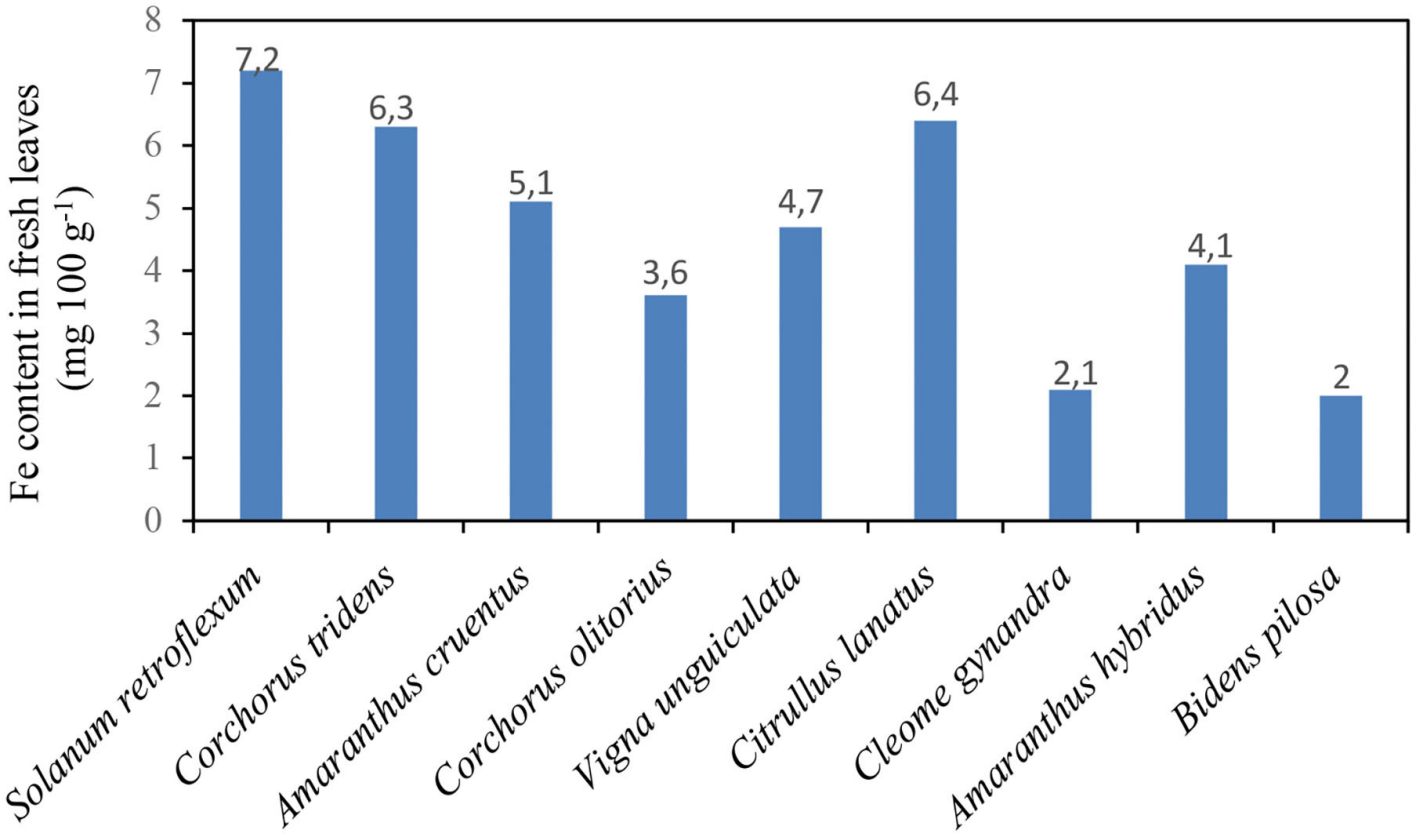
Iron content in *Solanum retroflexum* leaves compared with the other traditional leafy vegetables Source (15).

Figure 2 shows the iron (Fe) content in African nightshade species *S. retroflexum* (per 100 g fresh weight, FW) compared with other traditional vegetables in the Southern African region. The iron content in African nightshade was higher than that in other traditional vegetables in the Southern African region. A 100 g (FW) portion of African nightshade *S. retroflexum* contained 7.2 mg of iron, whereas other traditionally southern African vegetables, such as *C. tridens* (wild jute), *Amaranthus cruentus* (pigweed), *C. olitorius* (Jew's mallow), *V. unguiculata* (cowpea), *C. lanatus* (tsamma melon leaves), *C. gynandra* (spider flower), *Amaranthus hybridus* (cockscomb), and *Bidens pilosa* (black jack), contained 6.3, 5.1, 3.6, 4.7, 6.4, 2.1, 4.1, and 2.0 mg, respectively, in 100 g (FW) portion (7, 15).

Iron is regarded as an essential trace element for many bodily functions, such as biosynthesis of hemoglobin and activity of the central nervous system, and it also participates in the oxidation of macronutrients (e.g., carbohydrates, proteins, and fats) (16). The recommended daily intake (RDI) of iron is 8 mg day^−1^ for adults (17). Leaves of *S. villosum* were reported to contain 12 mg 100 g^−1^ iron on dry weight (DW) basis (15, 18). *S. villosum* contained higher iron levels than *C. gynandra* (spider plant) (48.6 mg 100 g^−1^) and *A. cruentus* (Madiira AM 38) (52.66 mg 100 g^−1^) at level-three maturity stages (7, 15, 18). This information suggests the best harvesting time for optimal Fe levels to the consumer.

The calcium content in a 100 DW portion of *S. villosum* is 442 mg, which was higher than the amount reported in *S. retroflexum* (199 mg), *C. maxima* (pumpkin leaves) (177 mg), and *B. pilosa* (black jack) (162 mg). Calcium is necessary for the maintenance of strong bones and teeth; 99% of calcium in the human body is used for this function (19). The RDI of calcium is 1,200 mg for adults (20).

The magnesium content in a 100 DW portion of *S. retroflexum* (92 mg) was reportedly higher than that of wild jute (80.9 mg), Jew's mallow (87 mg), cowpea (62 mg), pumpkin leaves (67 mg), tsamma melon leaves (59 mg), spider flower (76 mg), and black jack (79 mg) (7, 15, 21). Manganese content in *S. retroflexum* (2,080 μg 100 g^−1^) was higher than that found in spider flower (580 μg 100 g^−1^), tsamma melon leaves (760 μg 100 g^−1^), pumpkin leaves (540 μg 100 g^−1^), Jew's mallow (790 μg 100 g^−1^), cockscomb (4.1 μg 100 g^−1^), and black jack (2.5 μg 100 g^−1^) (21). Magnesium functions as a cofactor for more than 300 enzymatic reactions, and the RDI of magnesium is 420 mg for adult males and 320 mg for adult females (22).

The copper content in *S. retroflexum* (0.16 mg 100 g^−1^) was higher than that found in cowpea (0.14 mg 100 g^−1^). Copper is an essential mineral needed for growth and multiple functions, such as cardiovascular integrity, lung elasticity, neovascularization, neuroendocrine activity, and iron metabolism (23). The WHO guidelines advise 30 μg kg^−1^ of body weight per day^−1^, which is about 2 mg per day^−1^ for the average adult (23).

When compared with the commonly consumed exotic leafy vegetables, spinach, lettuce, kale, mustard green, rapini (broccoli raab), and Swiss chard greens (3), *S. retroflexum* (15) revealed higher levels of calcium, magnesium, iron, and copper (Table 1).

**Table 1:** Minerals and protein in African nightshade (per 100 g fresh weight) compared with other commercial vegetables.

	**Protein (g)**	**K (mg)**	**P (mg)**	**Ca (mg)**	**Mg (mg)**	**Fe (mg)**	**Cu (mg)**	**Zn (mg)**
*S. retroflexum*	6	257	36	199	92	7.2	0.16	0.56
*S. villosum*	4.6			442		12		
Spinach	2.86	558	49	99	79	2.71	0.13	0.53
Lettuce	0.9	141	20	18	7	0.41	0.025	0.15
Kale	2.92	348	55	254	33	1.6	0.053	0.39
Mustard green	2.35			118		1.69		
Rapini (broccoli raab)	3.17	196	73	108	22	2.14	0.042	0.77
Swiss chard greens	1.8	379	46	51	81	1.8	0.366	0.36

*Source: (8)*.

### Macronutrients

African nightshade *S. retroflexum* contained higher amounts of proteins (6%) than other African traditional vegetables (Table 1), such as *C. maxima* (pumpkin leaves) (4.24%), *A. cruentus* (pigweed) (3.49%), *C. olitorius* (Jew's mallow) (5.19%), *V. unguiculata* (cowpea) (4.7%), *C. maxima* (pumpkin leaves) (2.9%), *C. lanatus* (tsamma melon leaves) (3.5%), *C. gynandra* (spider flower) (5%), *B. pilosa* (black jack) (6%), and *A. hybridus* (cockscomb) (5%) (7, 15). Conversely, *S. villosum* showed lower levels of proteins (4.6%) than *S. retroflexum*, but similar levels to pumpkin and cowpea leaves (7, 15, 18).

In addition, a 100 g portion of *S. retroflexum* and *S. villosum* leaves had a sugar content of 1.02 and 1.15 g, respectively. For fat content, *S. retroflexum* contained monounsaturated fatty acids and omega 3 and 6 fatty acids at concentrations of 2.61, 0.33, and 0.63 g in a 100 g portion, respectively (24). Most importantly, due to its higher levels of proteins and iron, African nightshade is an important nutritional source for African people (11).

## Composition of Phytochemical Components in Different African Nightshade Species

Phytochemicals, plant-based non-nutritive compounds that contribute toward biological activity, aid in protecting the body against non-communicable diseases (25). An ~120 g portion of fruits or vegetables provides 100 different phytochemicals (26), among which phenolic compounds are the most abundant functional compounds. Aerial parts, especially the leaves, of different nightshade plants, *S. nigrum, S. scabrum, S. americanum, S. villosum*, and *S. retroflexum*, predominantly contain chlorogenic acid (caffeoylquinic acid), which belongs to the group of hydroxycinnamic acids (8) and is an ester of caffeic and quinic acids. Chlorogenic acid is a pronounced phenolic compound in *Amaranthus* leaves, including the red (*Amaranthus tricolor*) and green (*Amaranthus lividus*) genotypes (27), and a major phenolic compound in lettuce. Caffeoylmalate was detected in *S. scabrum* and *S. retroflexum* (27, 28). Kaempferol glycoside derivatives in *S. scabrum* leaves were mainly kaempferol-3-diglucoside, kaempferol-3-glucosylrhamnogalactoside, and kaempferol-3-rhamnosyl-rhamnogalactoside (isomers) (28) (Table 2). It was found that *S. retroflexum* leaves contained the following kaempferol derivatives: kaempferol-3-*O*-sinapoyldihexoside-hexoside, kaempferol-3-*O*-rutinoside, and kaempferol-dihexoside (28) (Table 2). The concentration of kaempferol derivatives in fresh *S. retroflexum* was at lower concentrations than that in *S. scabrum* (Table 2). Isorhamnetin-*O*-hexoside and rutin were found in *S. retroflexum* leaves (28). *S. scabrum* leaves contained mainly non-acylated quercetin glycosides, such as quercetin-3-neohesperidoside-7-glucosylrhamnoside (isomers), quercetin-3-rutinoside-7-rhamnosylglucoside, quercetin-3-galactorhamnoside, quercetin-3-rhamnosylgalactoside, quercetin-3-pentosylglucoside, and quercetin-3-pentosylrutinoside (28); conversely, quercetin-3-*O*-xylosyl-rutinoside was only detected in *S. retroflexum* leaves (28). Among the 11 phenolic compounds found in *S. retroflexum* leaves, rutin was the predominant compound.

**Table 2:** Concentration of different kaempferol derivatives present in *Solanum scabrum* and *Solanum retroflexum* species.

	**Kaempferol-3-diglucoside**	**Kaempferol-3-glucosylrhamnogalactoside**	**Kaempferol-3-rhamnosyl-rhamnogalactoside (isomer 1)**	**Kaempferol-3-rhamnosyl-rhamnogalactoside (isomer 2)**	**Total amount of kaempferol derivatives**
			**mg g**^**−1**^		
*Solanum scabrum*	0.057	0.043	0.083	0.040	0.223
			**mg g**^**−1**^		
	Kaempferol-sophoroside-hexoside	Kaempferol-3-*O*-hydroxyferuloyl-trihexoside	Kaempferol-3-*O*-hydroxyferuloyl diglucoside	Kaempferol-dihexoside	
*Solanum retroflexum*	0.051	0.0041	0.028	0.020	0.098

*Source: (28, 29)*.

Vitamin E content in *S. nigrum* and *S. scabrum* varied from 92.0 to 229.7 μg g^−1^ and from 90.4 to 192.5 μg g^−1^ on DW basis, respectively (8). *S. nigrum* (PI 312110, USDA) contained the highest amount of vitamin E of all African nightshade species (8), and *S. scabrum* (SS 04.2, World Vegetable Center [WAC] East and Southern Africa, Arusha, Tanzania) contained the lowest (8). In *S. americanum* (PI 268152, USDA) and *S. villosum* (Grif 16939, USDA), the vitamin E content was 145.5 and 114.3 μg g^−1^, respectively (8).

Total carotenoid content in *S. scabrum* leaves ranged from 586 to 691 μg g^−1^ on DW basis and 0.733 μg g^−1^ in *S. retroflexum* leaves on FW basis (8, 30, 31). The β-carotene content in the leaves of *S. nigrum* and *S. scabrum* species differed from 28.1 to 141.7 μg g^−1^ DW and from 55.1 to 96.0 μg g^−1^ DW, respectively (8). *S. villosum* (Grif 16939, USDA) reportedly contained the highest total carotenoids of 138.1 μg g^−1^ DW, whereas the lowest amount of 65.2 μg g^−1^ DW was found in *S. scabrum* (SS 04.2, WAC) (8).

African traditional leafy vegetables are rich in vitamin A and meet more than 75% of the recommended dietary allowance (RDA) (15). The vitamin A content in African nightshade (422 μg retinol activity equivalent, RAE) is greater than that in Jew's mallow (329 μg RAE), pumpkin leaves (325 μg RAE), and tsamma melon leaves (375 μg RAE) (15, 32).

Based on Yuan's et al. (8) findings, vitamin E, and total phenols contributed toward the antioxidant property [2,2′-azino-bis(3-ethylbenzothiazoline-6-sulfonic acid), ABTS] of the accessions of African nightshade *S. nigrum* PI 312110 and PI 381290, obtained from the USDA collection. *S. scabrum* (BG 16, Nduruma, WAC) showed the highest ABTS activity of 25 Trolox equivalent antioxidant capacity (TEAC) mg g^−1^ DW (8). Different African nightshade species from the USDA collection showed ABTS activity, on DW basis, in the following order: *S. scabrum* (25.00 TEAC mg g^−1^; BG 16, Nduruma), >*S. americanum* (24.81 TEAC mg g^−1^; PI 268152, USDA), >*S. nigrum* (23.93 TEAC mg g^−1^; PI 381290, USDA), ≥*S. nigrum* (23.45 TEAC mg g^−1^; PI 312110, USDA), >*S. scabrum* (22.46 TEAC mg g^−1^; SS 49 Olevolosi, WVC), *S. scabrum* (21.36 TEAC mg g^−1^; SS 52, WVC), ≥*S. scabrum* (21.26 TEAC mg g^−1^; Grif 14198, USDA), >*S. scabrum* (19.14 TEAC mg g^−1^; BG-29, WVC), >*S. nigrum* (18.34 TEAC mg g^−1^; PI 381289, USDA), >*S. scabrum* (17.92 TEAC mg g^−1^; Ex Hai, WVC), >*S. scabrum* (16.22 TEAC mg g^−1^; PI 643126, USDA), and >*S. nigrum* (15.49 TEAC mg g^−1^; PI 306400, USDA) (8).

Phenolic compounds positively correlated with antioxidant activity (33). Phenolic compounds participate in the antioxidant activity due to their redox properties, predominantly adsorbing, and neutralizing free radicals, quenching singlet and triplet oxygen, or decomposing peroxides (33). Current consumer trend is to replace synthetic antioxidants with natural dietary antioxidants for health benefits. Vitamin E (tocopherols) is important for disease prevention by preventing the breakdown of polyunsaturated fatty acids in membrane lipids and alleviating the oxidative stress (34). Lipophilic antioxidant ABTS activity demonstrated a strong correlation with tocopherols (35). However, correlations need to be established between the antioxidant activity and total phenolic content in different accessions of African nightshade.

## Impact of Irrigation on Minerals and Phytochemicals in African Nightshade

Minerals, such as Ca, Mg, K, and Mn, increased during severe stress in irrigation treatment at 30% field capacity, whereas P, Fe, and Zn content was the highest at 90% field capacity (28). Many developing countries adopt intercropping systems for effective use of the land, improving the productivity. The intercropping approach is popular among smallholder farmers (36). It can modify the nutritional composition in the plant parts, including the leaves; however, it did not significantly favor the accumulation of minerals in leaves in *S. scabrum* (28). The hydroxycinnamic acid derivatives, 3-caffeoylquinic acid, 5-caffeoylquinic acid, 4-caffeoylquinic acid, and caffeoylmalate, were significantly affected by the irrigation levels but not by the intercropping with *Brassica carinata* leaves (28); likewise, sinapoylmalate and sinapic acid were affected by irrigation. The irrigation treatments significantly affected the concentration of kaempferol-3-diglucoside in leaves (28). Ngwene et al. (28) confirmed that agronomy practices, such as intercropping, late-season drought, or irrigation management, can be adopted as a strategy to boost the levels of some health-related phytochemicals to benefit rural people and food manufacturers. The concentration of chlorogenic acid in *S. retroflexum* leaves (1.04 μg g^−1^) was less than that in pak choi (2.03 μg/g), salad spinach (1.59 μg g^−1^), red amaranth (9.06 μg g^−1^), and green amaranth (15.34 μg g^−1^) (37). Chlorogenic acid was mostly detected in *S. nigrum, S. scabrum*, and *S. villosum*; however, in some cases, chlorogenic acid was either detected in trace amounts or not detected, depending on the location (8). Quercetin-glucosyl-rhamnosyl-galactoside was detected at higher intensities in *S. nigrum, S. scabrum*, and *S. americanum*, but again not in all locations (8).

The other important phytochemicals in leafy vegetables are carotenoids. Low irrigation favored an increase in carotenoid accumulation in intercropped *S. scabrum*. The β-carotene and lutein contents in *S. scabrum* leaves increased when the irrigation treatment simulated drought treatment (30% water holding capacity, WHC) (28). The temperature variation during cold winter months and hot summer months did not show significant variation in total carotenoid content in *S. retroflexum* leaves (27).

## Impact of Postharvest Processing and Changes in Phytochemicals in African Nightshade

Postharvest processing (e.g., drying) and food preparation methods (e.g., cooking or fermentation) have a significant influence on the maintenance of phytochemical content of vegetables (38). Methods of postharvest processing can modify the composition of functional compounds in green leafy vegetables (29). Postharvest drying increased the income generation from traditional vegetable functional food. Global functional foods' market size is rising and expected to increase in 2025 to US$ 275.77 due to the increasing consumer appeal for nutritional and fortifying food additives (39). Traditionally adopted drying methods do not meet the requirement of homogenous quality standards; therefore, the installation of cost-effective solar dryers enables subsistence farmers to perform the postharvest drying at 50°C (30); this is a controlled drying method to prevent the depletion of vitamins and functional compounds.

**Figure 3:**
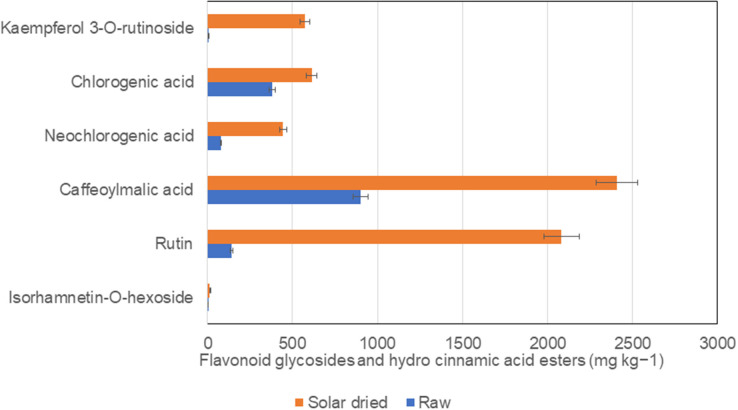
Comparison of phenolic components in solar dried and raw *S. retroflexum* leaves from Venda, Limpopo, South Africa. Source (24).

Solar drying of *S. retroflexum* leaves at 50°C increased the total carotenoid content by 40%, compared with the raw leaves. Similarly, phenolic metabolites, caffeoylmalic acid, rutin, and kaempferol-3-*O*-rutinoside revealed a remarkable increase in solar dried leaves. Chlorogenic and neochlorogenic acids substantially increased in solar dried *S. retroflexum* leaves (Figure 3), and antioxidant activity (FRAP) was enhanced compared with raw leaves (30). However, the temperature during the drying process plays a vital role in the chemical transformation of biochemical metabolites. Higher temperatures accelerate oxidation reactions, which can negatively affect the concentration of phenolic compounds (40). Loss of total polyphenols due to hot air drying have been reported previously (20).

The solar dried functional powder of *S. retroflexum* leaves contains 17.50 g carbohydrate in a 100 g portion; a lower carbohydrate content correlated with a lower calorific content 1,118.67 kJ 100 g^−1^ (267.36 cal) (Table 3) (30), the protein content is 32.91 g 100 g^−1^, slightly higher than the solar dried cowpea leafy vegetable (*V. unguiculata* L.) (29.40 g 100 g^−1^) and moringa leaves (*Moringa oleifera*) (28.09–28.99 g 100 g^−1^) (30, 41), and total dietary fiber is 28.81 g 100 g^−1^ (30). The solar dried *S. retroflexum* leaf powder is low in sodium, but the 100 g portion meets the daily requirement of potassium, calcium, and zinc intake; the iron and magnesium contents were ~8- and 2-fold higher than the required amount per day, respectively (Table 3) (30).

**Table 3:** Minerals in African nightshade (*S. retroflexum*) powder.

**Minerals**	**Quantity**	**Nutrition information**
Sodium (Na)	0.063 g 100 g^−1^	1.3 g day^−1^ AI [16]
Potassium (K)	4.9 g 100 g^−1^	4.7 g day^−1^ AI [16]
Iron (Fe)	60.6 mg 100 g^−1^	8 mg day^−1^ RDA [16]
Calcium (Ca)	1.400 mg 100 g^−1^	1,200 mg day^−1^ AI [16]
Magnesium (Mg)	556 mg 100 g^−1^	350 mg day^−1^ EAR [16]
Zinc (Zn)	14 mg 100 g^−1^	11 mg day^−1^ RDA [16]

*Source: (24)*.

*RDA, recommended dietary allowance; AI, adequate intake; EAR, estimated average requirement*.

In developing a low calorie, meal replacement product, solar dried functional powder of *S. retroflexum* leaves were utilized as the main ingredient. The final product contained 32.8 g of protein, 12.9 g of dietary fiber, 40 g of total sugar, 40.8 g of carbohydrate, 5.1 g of fat, and 1.4 g monounsaturated fatty acids (Table 4) with 369 cal in a 100 g portion (24). The protein content of African nightshade protein shake meets approximately half the daily requirement, but the available carbohydrate levels are much higher than those prescribed for daily intake (24). Low sodium content intake is preferred by those who suffer from high blood pressure and kidney problems; however, sodium is an important intracellular and extracellular cation that facilitates the regulation of plasma volume and acid–base balance during nerve and muscle contraction (42).

**Table 4:** Proximate analysis of the African nightshade (*S. retroflexum*) protein shake powder.

**Proximate composition**	**Quantity** **per 100 g DW**	% daily intake^*^ per serving (100 g powder)
Energy	1,544 kJ	18
	369 cal	
Protein	32.8 g	66
Total fat	5.1 g	7
Saturated fatty acids	1.3 g	5
Monounsaturated fatty acids	1.4 g	
Polyunsaturated fatty acids	2.4 g	
Trans fatty acids	<0.01 g	
Total sugar	40 g	44
Available carbohydrate	40.8 g	13
Total dietary fiber	12.9 g	43
Ash	3.4 g	n/a
Moisture content	5.0 g	n/a

*Source: (24)*.

* *Percentage daily intakes are based on an average adult diet of 8,700 kJ. Your daily intakes may be higher or lower depending on your energy needs; n/a, not applicable*.

Phenylalanine is an essential amino acid, which acts as a precursor of the amino acid tyrosine. Phenylalanine content in African nightshade powder was 308.7 mg g^−1^ (Table 4). It is generally recognized as “safe” by the Food and Drug Administration FDA (43). There are unlikely side effects reported at supplement doses of 50–100 mg per kg of body weight (44); therefore, it is possible for patients with amino acid metabolism disorder (phenylketonuria) to avoid the intake of high phenylalanine-containing meals (45). The African nightshade protein shake powder also contains aspartic acid (53.3 mg g^−1^), a non-essential amino acid used in building proteins; other plant sources of aspartic acid are avocado and asparagus. A serving size of 0.83 g of avocado provides 220 mg of aspartic acid, whereas a comparative portion of the protein shake powder contains 44 mg. Similarly, asparagus contains 5.08 mg g^−1^ of aspartic acid, which is much lower than the amount present in African nightshade powder (53.3 mg g^−1^; Table 5) (3). Another important non-essential amino acid in African nightshade powder is glycine, a powerful antioxidant with many health benefits.

**Table 5:** Amino acid profile of the African nightshade (*S. retroflexum*) protein shake powder.

**Amino acid components**	**Amount** (–H_**2**_O; mg g^**−1**^)^*^ Aver.	Amount (mg g^**−1**^)^**^ Aver.
Histidine	14.1 ± 0.3	17.0 ± 0.3
Serine	23.4 ± 0.6	26.1 ± 0.7
Arginine	10.2 ± 0.2	13.4 ± 0.2
Glycine	30.2 ± 1.1	34.9 ± 1.3
Aspartic acid	46.8 ± 1.5	53.3 ± 1.7
Glutamic acid	10.3 ± 0.2	12.2 ± 0.2
Threonine	10.7 ± 0.3	13.5 ± 0.3
Alanine	13.6 ± 0.3	16.1 ± 0.4
Proline	19.9 ± 0.7	22.7 ± 0.8
Lysine	8.4 ± 0.5	9.4 ± 0.5
Tyrosine	2.7 ± 0.3	3.1 ± 0.3
Methionine	14.3 ± 0.2	16.9 ± 0.3
Valine	13.6 ± 0.2	15.8 ± 0.3
Isoleucine	24.5 ± 0.5	28.4 ± 0.5
Leucine	16.3 ± 0.3	18.3 ± 0.3
Phenylalanine	265.9 ± 7.1	308.7 ± 8.2
Total	278.7	325.3

*Source: (24)*.

* *Calculation based on amino acid residue mass in protein (molecular weight minus H_2_O)*.

** *Calculation based on free amino acid molecular weight*.

Processing African nightshade leaves into powder is a preliminary step in the formulation of instant soups or meals. Evaluations indicated that this product will provide an instant, easy to handle and prepare meal, high in nutritional and sensory quality (46, 47).

### Fermentation—A Traditional Food Processing Technique

Fermentation is a traditional food processing technique, adopted as a preservation technique in Africa, performed with the objective of increasing food safety and make food more edible and appealing in terms of sensory properties, by improving flavor and aromas (32, 48). A frequently reported main effect of lactic fermentation is the improvement of the bioavailability of nutritional components. A popular fermented food on the African continent is *Kawal*, especially in Sudan, produced by spontaneous fermentation of leguminous leaves (49–51). *Bacillus* spp. and *Lactobacillus plantarum* are involved in fermentation, but most antinutritional factors, especially phytic acid content, decreased during the process. Fermentation of African nightshade (*S. scabrum*) and cowpea leaves, using *L. plantarum* and *Leuconostoc mesenteroides* ssp. *mesenteroides*, for 48 h reduced the pH and inhibited the growth of foodborne pathogens, such as *Listeria monocytogenes* and *Salmonella enterica* Enteritidis. Likewise, fermentation of African kale leaves (*B. carinata*) with *L. plantarum* BFE 5092 and *Lactobacillus fermentum* BFE 6620 starter strains inhibited the growth of *L. monocytogenes, S*. Enteritidis, and other enterobacteria, while maintaining appreciably the concentration of vitamin C (35 mg 100 g^−1^) in the fermented product (32). Oguntoyinbo et al. (32) demonstrated that controlled fermentation is a promising method to reduce food spoilage and extend shelf life and food safety.

Fermentation of African nightshade *S. scabrum*, using 3% salt–sugar solution with *L. plantarum* BFE 5092 and *L. fermentum* BFE 6620 as starter cultures, had a greater impact on the microbial profile of the fermented product due to the rapid and stable decline of pH and production of lactic acid (52). The fermented product retained substantial levels of vitamins B1, B2, and C, which are sufficient to supplement the RDI and improve the sensory attributes, color, taste, and aroma (52).

**Figure 4:**
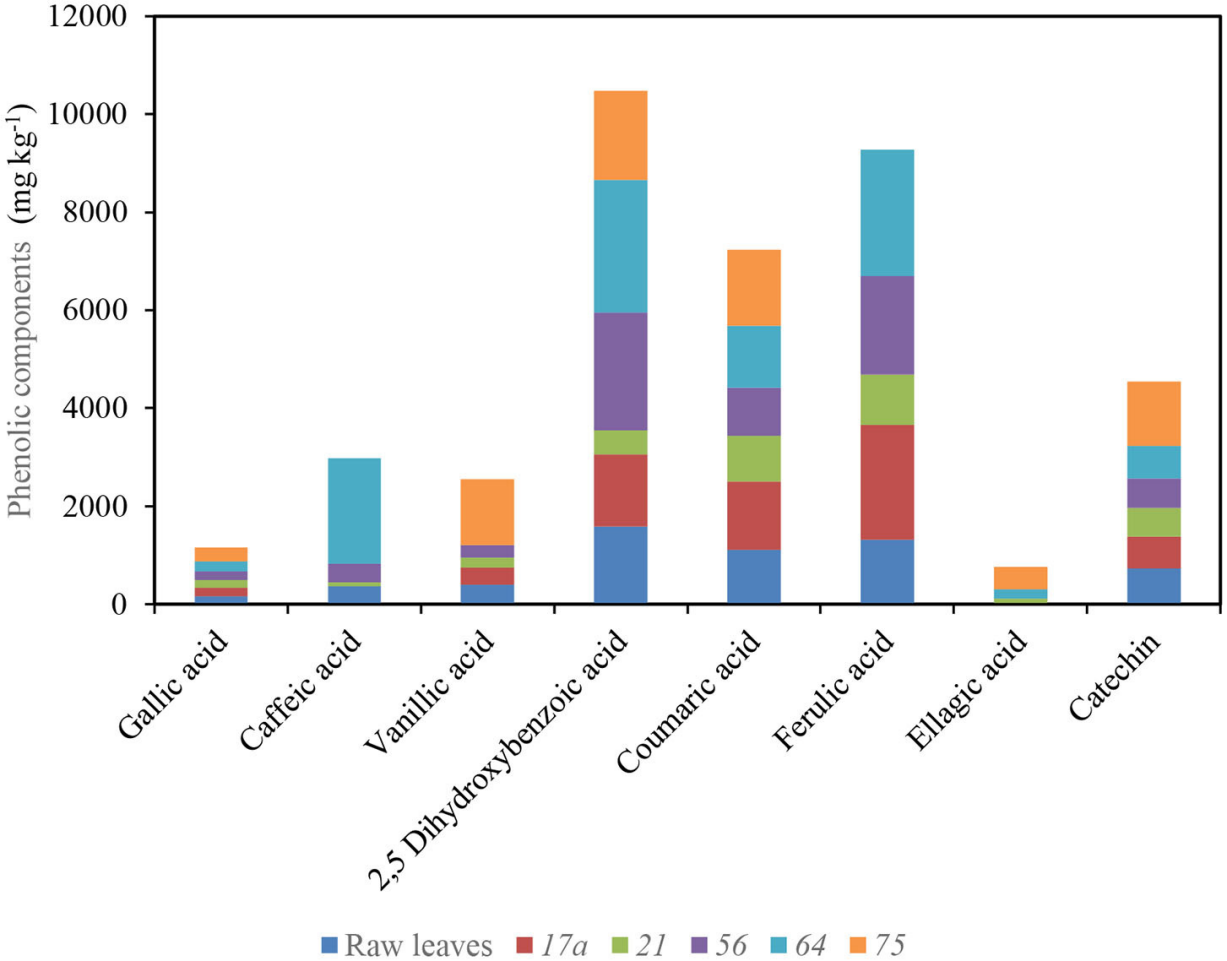
Impact of fermentation with different lactic acid bacterium strains on different phenolic components in African nightshade (*S. retroflexum*) leaves (75 *L. plantarum*, 21 and 64 *Weissella cibaria*, and 56 *Leuconostoc pseudomesenteroides*). Source (53).

*L. plantarum* 75 enhanced the functional potential of nightshade leaves and improved the bioavailability of phenolic compounds including phenolic acids (gallic, vanillic, 2,5-dihydroxybenzoic, coumaric, ferulic, and ellagic acids) and flavonoids (catechin, quercetin, and luteolin) in the fermented product of *S. retroflexum* (Figure 4) (53). However, caffeic and ferulic acids were not detected in African nightshade (*S. scabrum*) after fermenting with *L. plantarum* 75, possibly due to the different types of microbial metabolism and transformation of the phenolic compounds, which are predominantly determined by the different enzyme systems involved, irrespective of the species (53). Simultaneously, ferulic acid could have been reduced to dihydroferulic acid, whereas caffeic acid possibly metabolized to vinylcatechol, ethylcatechol, or dihydrocaffeic acid (54). Degrain et al. (53) concluded that *L. plantarum* 75 fermentation improved the extraction of phytochemical components in nightshade leaves and reduced carbohydrate content and calculated energy of the final product (53).

As with solar drying, fermentation of *S. retroflexum* with *L. plantarum* 75 improved the antioxidant activity compared with raw leaves (53); flavonoids containing multiple hydroxyl groups mostly have higher antioxidant activities. Many studies have underlined the beneficial health effect of antioxidant-rich foods, such as reducing the risk of non-communicable diseases and premature aging (55). Similarly, *S. scabrum* leaves fermented with *L. plantarum* BFE 5092 and BFE 6620 in 2.1 L of a 2.5% brine solution (containing 3% salt and 3% sugar) at 25°C for 144 h revealed an increase in the concentration of caffeoylquinic acid isomers, sinapoylmalate, kaempferol-3-diglucoside, quercetin-3-pentosylrutinoside, sinapic acid, quercetin-3-rutinoside, and caffeoylmalate. The total polyphenols increased in concentration compared with the raw leaves, whereas quercetin-3-glucosyl-rhamnogalactoside and quercetin-3-rhamnosyl-rhamnogalactoside slightly reduced in the fermented leaves compared with the raw leaves (38). This is possibly due to the action of glycosyl hydrolases generated from the fermentation activity of lactobacillus strains on the conversion of flavonoid glycosides to the corresponding aglycones, which shows the higher enhancement of antioxidant activity and the biological bioactivity and benefit to the consumers (56). Coumaric acid levels after fermentation also reduced in *S. scabrum* leaves (38), possibly due to the coumaric acid acting as an external acceptor of electrons to gain one extra mole of ATP (57).

Proximate analysis of fermented African nightshade (*S. retroflexum*) leaves with *L. plantarum* 75 showed 2.51 g carbohydrate content in a 100 g serving portion. Dietary fiber, protein, fat, and sugar contents in a 100 g serving portion of fermented African nightshade vegetable product were 2.52, 3.82, 0.23, and <0.50 g, respectively. However, sodium content (231 g) was higher in the fermented nightshade vegetable product than in the solar dried powder of the same product (53). Sodium content is of great concern, and the daily limit of 2.4 g per day is due to an increase in the prevalence of chronic diseases, such as high blood pressure, which positively correlate to high salt intake (54).

### Household Cooking

Traditionally, the consumption of African nightshade leaves is after cooking, and the adoption of different cooking methods, such as a boiling and steaming, is to improve palatability and sensory properties. Cooking can enhance the bioavailability of phenolic components and the antioxidant activity (58); however, it can have deleterious effects on the nutrient composition and functional compounds and their bioavailability in different vegetables (59).

Traditional food preparation methods, including blanching, are widely adopted to improve the palatability and reduce the bitterness of African nightshade leaves. Blanching treatments (steam or cook in hot water using plain water or lemon juice) at 95°C for 5 min increased the concentrations of hydroxycinnamic acid derivatives (chlorogenic, neochlorogenic, and cryptochlorogenic acids) and caffeoylmalic acid (60). However, steam blanching in either water or lemon juice at 95°C for 5 min significantly improved the concentration of caffeoylmalic acid. In addition, cooking improved the antioxidant capacity of vegetables (58). The increase of chlorogenic acid concentration in *S. retroflexum* leaves during blanching treatments could be due to the formation of different caffeoylquinic acid isomers or the hydrolysis of dicaffeoylquinic acid (59). A similar significant increase in caffeoylquinic acid was reported in fried artichokes compared with raw and other cooking methods (60). Transesterification of caffeoylquinic acid is dependent on the pH of the food matrix, as well as temperature and time.

*S. scabrum* leaves boiled in water demonstrated an increasing trend in the levels of 3-caffeoylquinic acid, 5-caffeoylquinic acid, and 4-caffeoylquinic acid compared with the raw leaves. Boiling also reduced the levels of caffeoylmalate compared with the raw leaves and remarkably reduced the levels of quercetin-3-glucosylrhamnogalactoside, coumaric acid, quercetin-3-rhamnogalactoside, quercetin-3-rhamnosyl-rhamnogalactoside isomers, kaempferol-3-rhamnosyl-rhamnogalactoside, quercetin-3-pentosylrutinoside, and quercetin-3-rutinoside; sinapoylmalate, sinapic acid, kaempferol-3-diglucoside, and kaempferol-3-rhamnosyl-rhamnogalactoside were not detected in the cooked *S. scabrum* leaves (38). Non-acylated kaempferol diglucosides in broccoli demonstrated higher loss after boiling and minor loss after steaming, but during higher temperature heat treatments, it was expected that kaempferol-3-diglucoside would degrade to its monoglucoside or kaempferol; additionally, 4-O-position had a higher stability against deglucosilation than 3-O-position (58).

## Changes in Antinutritive Compounds in African Nightshade During Postharvest Processing

Some accessions of African nightshade leaves contain glycoalkaloids, which can cause health concerns. Among the five accessions of African nightshade, *S. nigrum* reportedly contains solasodine glycosides, including solamargine and solasonine, as in other plants belonging to the family Solanaceae, such as potatoes, tomatoes, and eggplants. Yuan et al. (8) reported the absence of glycoalkaloids in methanol leaf extracts of *S. scabrum* and *S. villosum*.

Based on previous reports (8), safe consumption of eggplants was allowed at glycoalkaloid levels ranging from 6.25 to 20.5 mg 100 g^−1^ FW. Higher levels of glycoalkaloids were detected in commonly consumed African nightshade leaves, which were confirmed as safe for consumption (8).

Steroidal saponins, mass of tigogeninas, were detected in *Solanum* spp. Tigogenin-5G is detected in most of the African nightshades spp. Dehydrodiosgenin-G-G-Rha-Rha and diosgenin-G-G-Rha-Rha are detected only in *S. nigrum* from the USDA collection PI 312110. Tigogenin-G-G-Rha-Xyl-Xyl is detectable in all *S. nigrum* (Kenya) from the USDA collection PI 306400, PI 381289, and PI 381290; *S. scabrum* SS 5, Ex Ha, SS 49, Olevolosi SS 04.2, BG 16, Nduruma BG-29, Grif 14198, and PI 643126; *S. americanum*; and *S. villosum*. Tigogenin-G-G-G is detected mainly in *S. nigrum* obtained from Kenya, USDA collection PI 30640, and in *S. villosum* (8). Tigogenin-3G-Xyl-G, tigogenin-5G, and tigogenin-GG-Rha-Xyl-Xyl are detected in *S. retroflexum* (30). The raw leaves contained 0.45 mg kg^−1^ of tigogenin and 0.56 mg kg^−1^ of tigogenin-GG-Rham-Xyl-Xyl, and solar drying increased the levels of tigogenin and tigogenin-GG-Rham-Xyl-Xyl to 70.54 mg kg^−1^ and 73.92 mg kg^−1^, respectively (30). Similarly, steam blanching, in water or lemon juice at 95°C for 5 min, increased the peak responses of the tigogenin-5-G, tigogenin-3G-Xyl-G, and tigogenin-GG-Rha-Xyl-Xyl, but the effect was greater in tigogenin-5-G (30). Tigogenin is an important raw material for pharmaceutical use and the synthesis of steroid drugs, demonstrating anti-inflammatory, and anti-diabetic activities (type 2 diabetics) (61).

The raw leaves of *S. retroflexum* contain other antinutritive compounds, such as tannins (55.4 mg 100 g^−1^), phytates (88 mg 100 g^−1^), and oxalates (87.5 mg 100 g^−1^). Hot water blanching and steam blanching treatments during food preparation help to significantly reduce the levels of these compounds (61).

Tannins, which are polyphenols, can prevent the availability of protein for absorption by forming complexes with proteins (61). Oxalates also prevent the absorption of dietary calcium by binding with Ca^2+^ (61, 62); furthermore, the insoluble calcium oxalates are stored in the kidney, causing “kidney stones.” The increased oxalate:calcium ratio >9:4 can affect Ca absorption negatively (63). Phytic acid chelates with Zn or phytates, binding with proteins making them unavailable for absorption (62).

## Functional Potential for Consumers' Diet

Inclusion of African nightshade, especially *S. retroflexum*, with the main staple food can improve protein, iron, and calcium levels, which will help improve people's health and well-being. It is also possible to use African nightshades as food and medicinal ingredients. The increased polyphenol compounds can contribute to antioxidant activity, and an increased intake of chlorogenic acid correlates with the reduced risk of type 2 diabetes mellitus (64). Available literature suggests that the chlorogenic acid suppresses postprandial hyperglycemia by inhibiting α-glucosidase similar to the α-glucosidase inhibitors, such as acarbose, miglitol, and voglibose (64). At the same time, chlorogenic acid modulates the glucose and lipid metabolism *via* the activation of adenosine monophosphate-activated protein kinase and stimulates glucose uptake in the skeletal muscle, which shows similar activity as anti-diabetic agents (65).

Solar dried or blanched African nightshade *S. retroflexum* leaves can be included as a functional ingredient or a functional food to manage type 2 diabetes in rural regions and as a promising potential for the food industry and food manufacturers. For health claims, and to popularize use as an ethnic food, future research on the biological activities of African nightshade leaves on anti-diabetic, anti-proliferative, or anti-inflammatory effects is necessary. Furthermore, *in vitro* results revealed the chemo-preventive properties in terms of anti-genotoxicity against the liver carcinogen aflatoxin B1 (AFB1) and antioxidant potential, at non-toxic concentrations, of the leaf extract of *S. scabrum* (38). The authors concluded that although the food preparation and processing methods affected the concentration of phytochemicals, the compositional changes could have acted positively in the observed antioxidant activity and chemo-preventive properties (38).

## Conclusions

African nightshade vegetables are rich in minerals and phytochemicals, and the adoption of different food processing or preparation methods can prevent postharvest losses during the supply chain to contribute to food security and reduce hidden hunger. Food processing or preparation methods improved the phytochemicals (functional compounds) in the African nightshade vegetables, and agronomy practices affected the nutritional properties. For all the USDA and WAC African nightshade collections, it is important to profile the mineral composition to select the best accession to benefit consumers. Based on the available literature regarding the antioxidant activity, the recommendation for growers is to use *S. scabrum* (BG 16, Nduruma, WAC); however, all the accessions need further testing to correlate with the biological actives in order to identify the suitable accession for commercial production and marketing.

## Author Contributions

DS obtained the funding for the program and conceptualized the research. AP formatted and validated the data. YS under the Australia–Africa partnership program, collaborated with data generation. RS research collaborator, proofread and edited the review. FR research collaborator under the SA-France bilateral program, assisted with the generation of some data and editing of the review. All authors contributed to the article and approved the submitted version.

## Conflict of Interest

The authors declare that the research was conducted in the absence of any commercial or financial relationships that could be construed as a potential conflict of interest.
